# Supplementary material for: Targeting Protein Translation in Melanoma by Inhibiting EEF-2 Kinase Regulates Cholesterol Metabolism though SREBP2 to Inhibit Tumour Development
Source: Int J Mol Sci. 2022 Mar 23;23(7):3481. doi: 10.3390/ijms23073481 (PMC8998919; doi:10.3390/ijms23073481)
Supplement: Supplementary file 1 [file ijms-23-03481-s001.zip › Supplementary Figures.pdf]

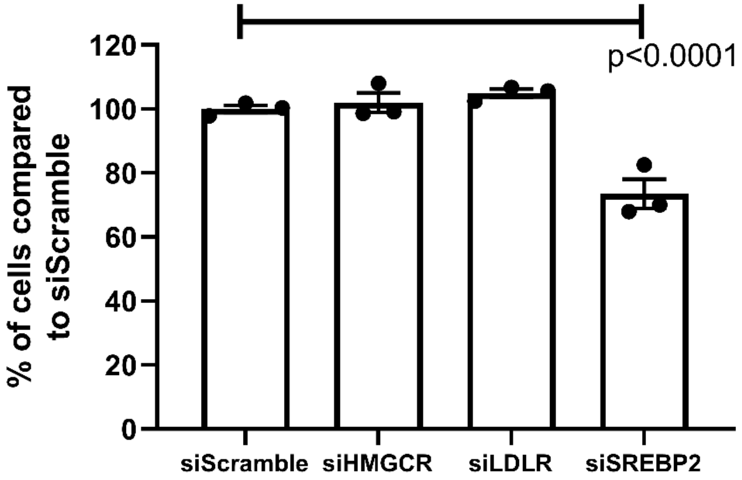

Figure S1 Effect of cholesterol pathway inhibition on melanoma cell proliferation. Genes regulating cholesterol uptake and synthesis were knocked down using siRNA and evaluated for effect on UACC 903 melanoma cell survival after 72 hours by MTS assay.

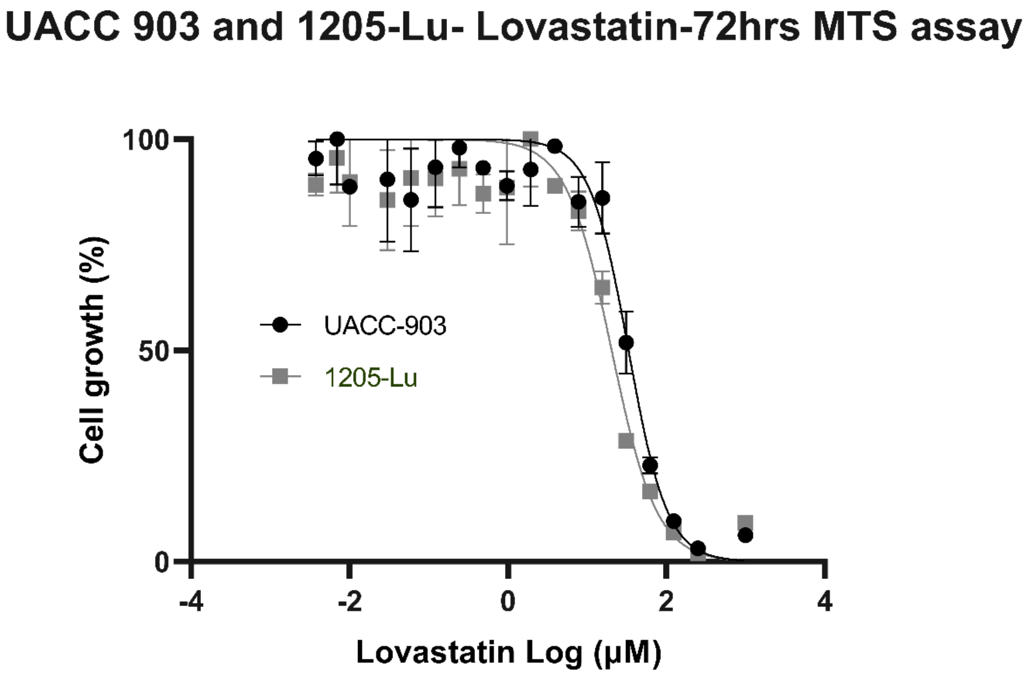

	UACC 903	1205 Lu
IC_{50}	33.36	20.43

Figure S2 Efficacy of Lovastatin in reducing melanoma cell survival by blocking cholesterol synthesis. Lovastatin was evaluated for its ability to reduce the survival of UACC 903 and 1205 Lu melanoma cells after 72-hour treatment. Values below the graph represent the IC_{50} concentrations in µM.

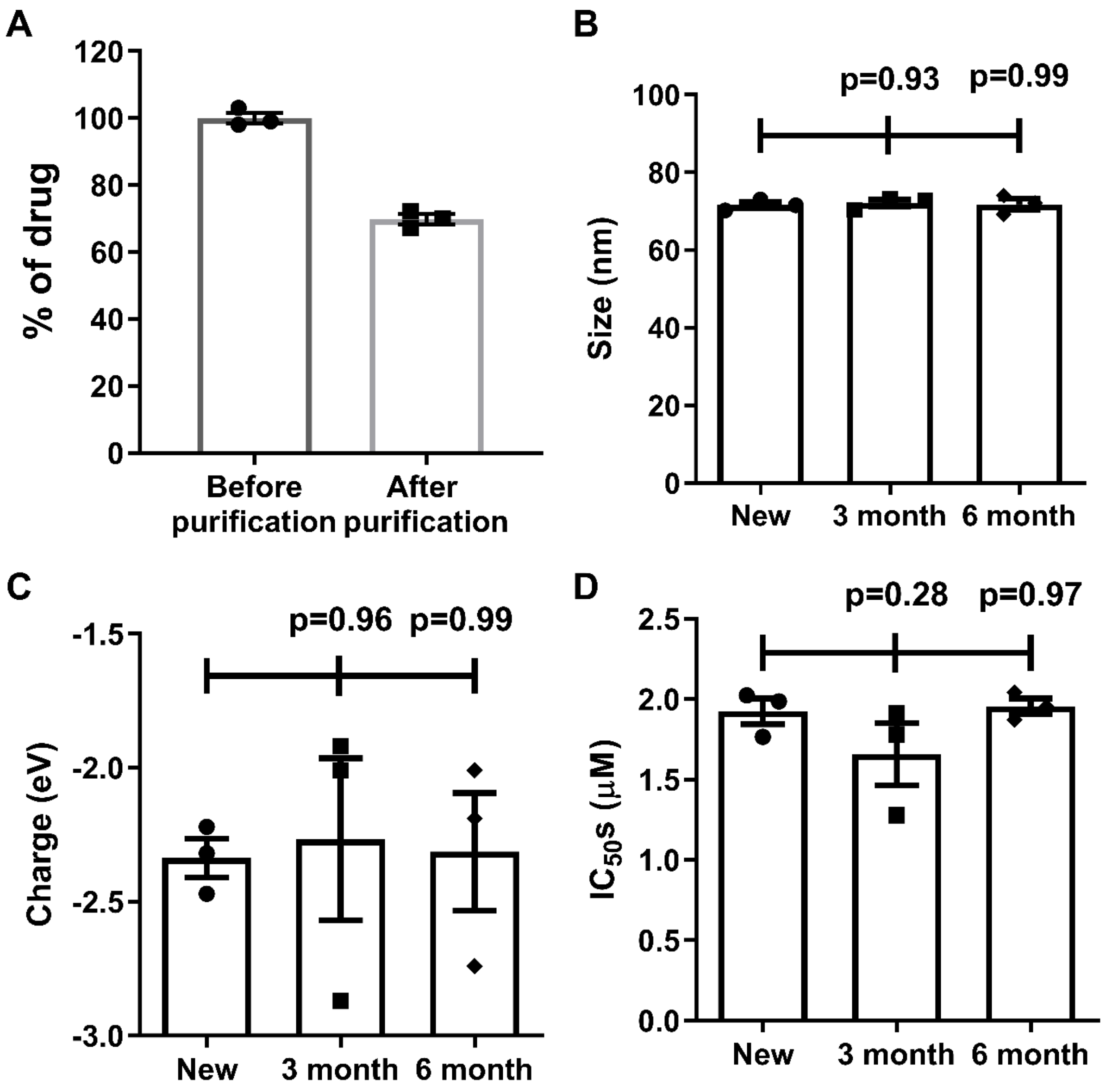

Figure S3 Development and characterization of the nanoliposomal formulation of NH125, called NanoNH125. NanoNH125 was manufactured with a 68% loading efficiency of NH125 in the nanoliposomes (3A). NanoNH125 is stable for at least 6 months when stored at 4˚C with no significant changes in size (3B) or charge (3C) or cell killing IC_{50}s (3D). Significance was compared to freshly prepared liposomes by one-way ANOVA followed by Dunnett's as post-hoc analysis.

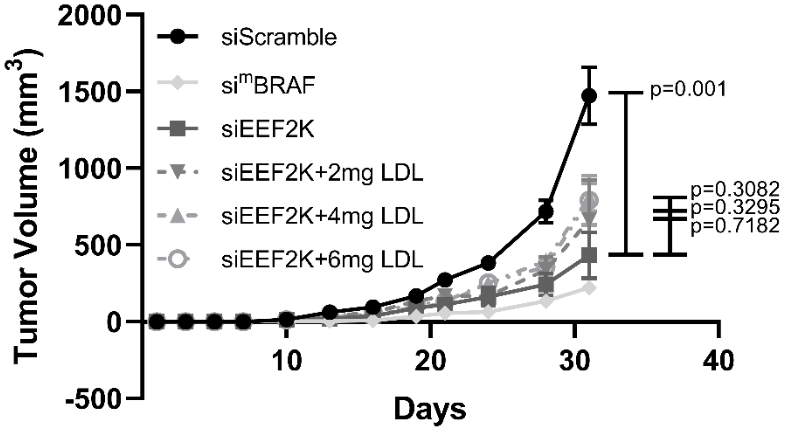

Figure S4 Efficacy of LDL supplementation for reversing siEEF2K-mediated tumor inhibition. Daily LDL supplementation at 2, 4, and 6 mg slightly reversed the effect of siEEF2K on UACC 903 tumor inhibition. P-values were not significant compared to siEEF2K inhibition.

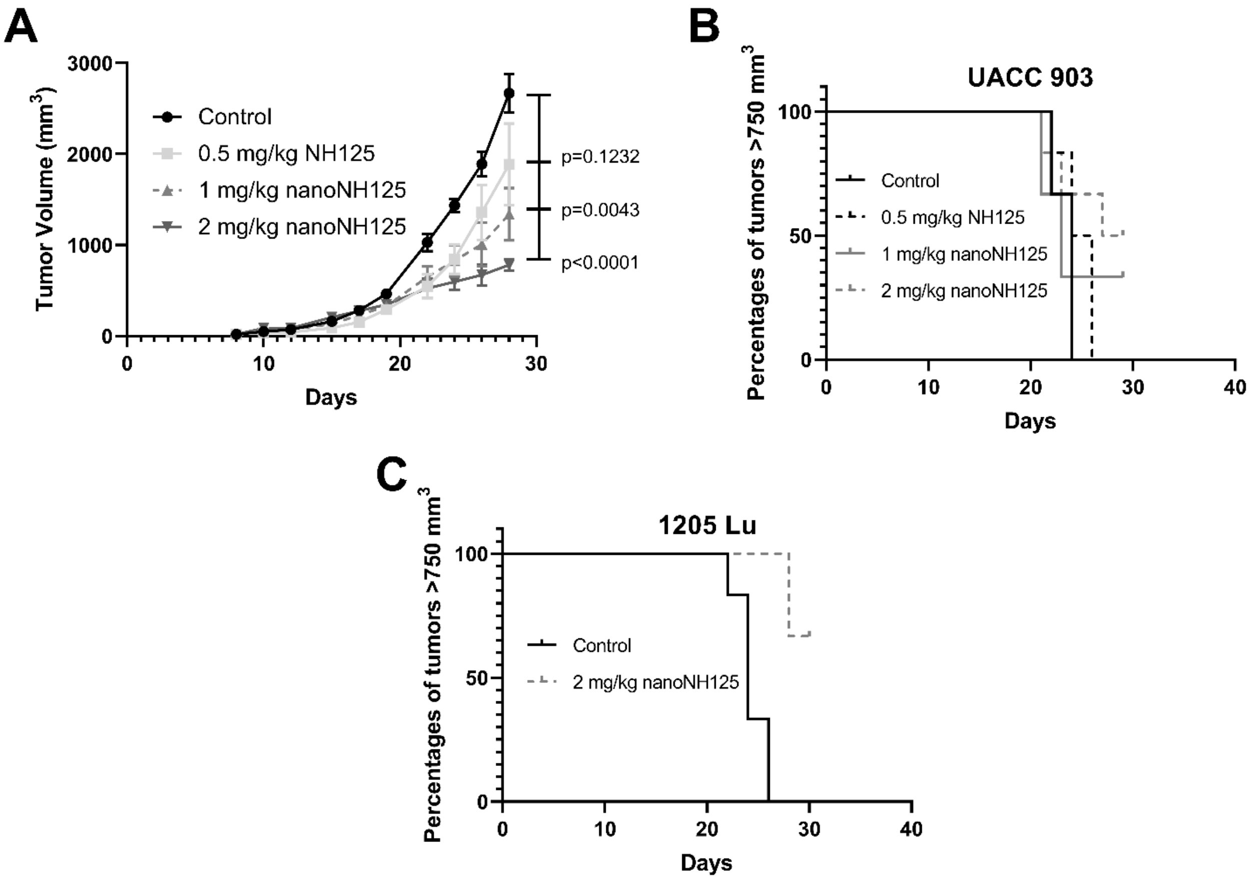

Figure S5 Comparison of tumor kinetics and survival with free NH125 versus NanoNH125 treatment. The tumor kinetics of 0.5 mg/kg NH125, 1 and 2 mg/kg NanoNH125 treatment groups were compared to a pooled control consisting of animal treated with DMSO and EL (5A). P-values are different than those in **Fig. 6** since controls (DMSO and EL) were pooled to obtain an unbiased assessment of inhibitory efficacy. A Kaplan-Meier curve showed the time required for UACC 903- (5B) and 1205 Lu- (5C) bearing mice to develop tumors over 750 mm^3.
