# Supplementary material for: Targeting Protein Translation in Melanoma by Inhibiting EEF-2 Kinase Regulates Cholesterol Metabolism though SREBP2 to Inhibit Tumour Development
Source: Int J Mol Sci. 2022 Mar 23;23(7):3481. doi: 10.3390/ijms23073481 (PMC8998919; doi:10.3390/ijms23073481)
Supplement: Supplementary file 1 [file ijms-23-03481-s001.zip › Table S1.pdf]

Daily dose (mg/kg body weight) for 7 days	NH125			NanoNH125		
	i.p. administration			i.v. administration		
	Mortality at day 7	Behavioral parameters indicating toxicity	Average % weight loss compared to control	Mortality at day 7	Behavioral parameters indicating toxicity	Average % weight loss compared to control
0.5	0/3	None	No loss	0/3	None	No loss
1	1/3	Hunched back; lethargic; distorted abdomen	12.1	0/3	None	No loss
2	3/3	-	-	0/3	None	No loss
4	-	-	-	0/3	Hunched back	1.2
8	-	-	-	3/3	-	-

Supplementary Table S1 Assessment of toxicity of NH125 versus NanoNH125 in mice. A 7-day repeated dose study was conducted for both NH125 and NanoNH125. NH125 was administered i.p. daily and NanoNH125 was administered i.v. daily at 0.5 to 8 mg/kg. Animal body weight, behavioral changes indicative of toxicity (hunched back, lethargy, distorted abdomen), and mortality were monitored. NH125 was toxic starting at 0.5 mg/kg while NanoNH125 only showed toxicity at 4 mg/kg.
